# Multimodal Prehabilitation in Head and Neck Cancer Patients Undergoing Surgery: A Feasibility Study

**DOI:** 10.1111/jhn.70047

**Published:** 2025-03-27

**Authors:** Lennaert C. B. Groen, Celine D. de Vries, Doriene C. Mulder, Freek D. Daams, Emma R. J. Bruns, Renée Helmers, Hermien W. H. Schreurs

**Affiliations:** ^1^ Department of Surgery Northwest clinics Alkmaar the Netherlands; ^2^ Department of Oral and Maxillofacial Surgery Northwest clinics Alkmaar the Netherlands; ^3^ Department of Surgery Academic University Medical Center location VU Amsterdam the Netherlands; ^4^ Department of Surgery Spaarne Hospital Haarlem the Netherlands

**Keywords:** head and neck cancer, prehabilitation, surgery

## Abstract

**Background:**

Head and neck cancer (HNC) incidence is increasing, and surgery is frequently indicated as curative treatment. Unfortunately, complications and long‐term functional impairment are common. Recent promising results of multimodal prehabilitation in colorectal cancer surgery show improved recovery and functional outcomes. The objective of this study is to assess the feasibility of multimodal prehabilitation, which is composed of high‐intensity training, a protein‐enriched diet, cessation of intoxications, mental support and speech support therapy, in HNC surgery.

**Methods:**

A feasibility study was conducted at a large teaching hospital, Northwest Clinics, Alkmaar, the Netherlands, between July 2022 and December 2023. The primary outcome was feasibility, defined as participation, dropout and adherence rate. The secondary outcome was functional capacity 6 weeks postoperatively.

**Results:**

The participation rate was 60% (30 of 50 patients), mainly limited due to the travel distance to the physiotherapist. A dropout rate of 7% was present, as two patients discontinued prehabilitation. Of the remaining 28 patients, 27 patients (96%) attended at least six sessions at the community physiotherapist practice. All functional tests increased by prehabilitation, with the 6‐min walking test being significant (*p* ≤ 0.05). Six weeks postoperatively, all but steep ramp tests remained higher than baseline.

**Conclusion:**

Feasibility of multimodal prehabilitation in HNC surgery patients in this study is limited by its participation rate of 60%. Addressing participation, a widespread network of oncologic physiotherapists or home‐based multimodal prehabilitation by an app could possibly potentiate participation. More studies are needed to assess the optimal form of multimodal prehabilitation in this challenging population.

## Summary

- This study describes the first results of the introduction of multimodal prehabilitation in head and neck cancer surgery patients.
- This study reports a low dropout and high adherence rate, challenged by a participation rate of 60%.

## 1 Introduction

Head and neck cancer (HNC) is a group of malignancies of which the majority occur in the oral cavity. It is the eighth and ninth most common type of cancer in men and women, respectively, with an incidence of 2.6% of all cancer types [1]. The incidence has increased in the Netherlands by 50% in the last three decades, potentially due to the rise in HPV‐related cancers [2]. The primary treatment for HNC is surgery, (chemo)radiation therapy or a combination of both depending on location and the stage [1].

Several challenges arise when treating HNC patients. A high comorbidity rate is observed, and many HNC patients have an extensive background of smoking and alcohol use [3]. Malnutrition, weight loss and muscle wasting are also common as the tumour impairs dietary intake [4]. As a consequence, HNC surgery shows relatively high complication rates and morbidity with long‐term decreased quality of life [3, 5, 6]. To reduce the impact of surgery on HNC patients, these potentially modifiable parameters could be optimised by multimodal prehabilitation. It comprises preoperative endurance and strength training, optimising nutrition with a high‐protein diet and supplementation of micronutrients, smoking cessation and psychological support [7]. Especially in colorectal cancer, recent evidence shows a reduction in complications and improved functional outcomes with decreased overall healthcare costs [8, 9]. In cancer surgery, unimodal prehabilitation was mainly offered in small sample sizes and heterogeneous groups, resulting in low‐quality evidence with limited overall certainty on the effect of prehabilitation [10, 11, 12, 13]. Studies in HNC, thus far, primarily focus on the effectiveness of nutritional interventions such as swallowing exercises reducing dysphagia and physical exercise programmes for patients undergoing radiotherapy and/or chemotherapy [14, 15, 16]. To our knowledge, there is no report on multimodal prehabilitation in HNC surgery patients to this date. This study aims to report the first results on the feasibility of multimodal prehabilitation in HNC surgery patients.

## 2 Methods

### 2.1 Study Design and Setting

A single‐centre feasibility study was conducted in a large regional hospital, Northwest Clinics, Alkmaar, the Netherlands. Recruitment of prehabilitation patients took place between July 2022 and December 2023. Data were prospectively collected in patients' electronic medical records after patient approval. All data was encoded and collected anonymously in an online database (Castor CDMS 2023.3). A 30‐day follow‐up was conducted. The ethical committee of the Academic Centre for Dentistry Amsterdam (ACTA) approved this study (2023‐52850). This study followed the reporting of observational studies in epidemiology (STROBE) guidelines for reporting data [17].

### 2.2 Participants

Patients were eligible if a combined mandibulectomy and neck dissection (commando procedure), transoral excision with sentinel node procedure (SNP) or a parotidectomy with neck dissection was indicated by a multidisciplinary tumour board. Patients were excluded from participation when they were paralysed, had advanced cognitive impairment or had renal insufficiency grade 3 or higher.

For a hospital to treat HNC patients in the Netherlands, the processing time requires that 80% of patients be treated within 30 days after the first consultation [18]. To maximise the time of participation in prehabilitation, patients were informed about the study during the first consultation, hence before definitive diagnosis and treatment strategy. After a reflection period and positive confirmation, informed consent was obtained.

### 2.3 Variables and Measurement of Both Groups

Patient characteristics were collected, including age, gender, intoxications, comorbidity by the Charlson Comorbidity Index [19], American Society of Anesthesiologists physical classification (ASA) score [20], metabolic equivalent for task units (METs) [21], cancer‐specific data and operative technique.

The primary outcome of this study is the feasibility of multimodal prehabilitation in HNC surgery patients, defined by the participation rate, dropout rate and adherence rate. The participation rate was calculated by patients willing to participate in this study, divided by all potentially eligible patients. Dropout rate was the percentage of patients who did not continue prehabilitation until surgery. Adherence rate was the percentage of supervised training followed until surgery. A minimum of six supervised training sessions was set, as there was limited processing time and the frequency of supervised training three times a week. Adverse events during prehabilitation were monitored.

Secondary outcomes include functional capacity before start of prehabilitation (baseline) compared to 6 weeks postoperatively. Other outcomes as the 30‐day complication rate according to the Clavien–Dindo classification [22] and Comprehensive Complication Index [23], 30‐day mortality, length of hospital stay and readmission rate were collected.

### 2.4 Multimodal Prehabilitation Measurements

Functional capacity was tested by two physiotherapists specialised in oncology (Master of Science), with experience in prehabilitation. Tests were conducted at their practice (Heerhugowaard and Julianadorp), close to our two outpatient clinic locations. Functional tests are composed of the one‐repetition maximum (1RM), sit‐to‐stand test (STS), 6‐min walk test (6MWT) and steep ramp test (SRT). For more details, see Supporting Information A.

Nutritional status was reviewed by a dietitian specialised in HNC patients, based on nutritional assessment, body mass index, body impedance analysis and the Patient‐Generated Subjective Global Assessment (PG‐SGA) [24]. For handgrip strength, the maximum score from 3 tests was recorded, and the average of both hands was considered appropriate [25]. One week after the start of prehabilitation, the dietitian conducted a telephone consultation to assess the diet and adherence based on a food diary and logbook. Additionally, a final evaluation of adherence to the diet was conducted 1 day before surgery.

At consecutive measurement moments, mental distress was assessed by the Distress Thermometer [26]. Patients aged 70 years or older were screened for frailty by a geriatrician and on indication a comprehensive geriatric assessment was performed.

An overview of the prehabilitation measurement points scheme is presented in Supporting Information B.

### 2.5 Bias and Study Size

As described in section participants, consecutive patients were evaluated for eligibility to this study, minimising selection bias.

A convenient sample size of 30 patients was deemed appropriate to assess the feasibility of multimodal prehabilitation.

### 2.6 Intervention: Multimodal Prehabilitation

**Figure 1:**
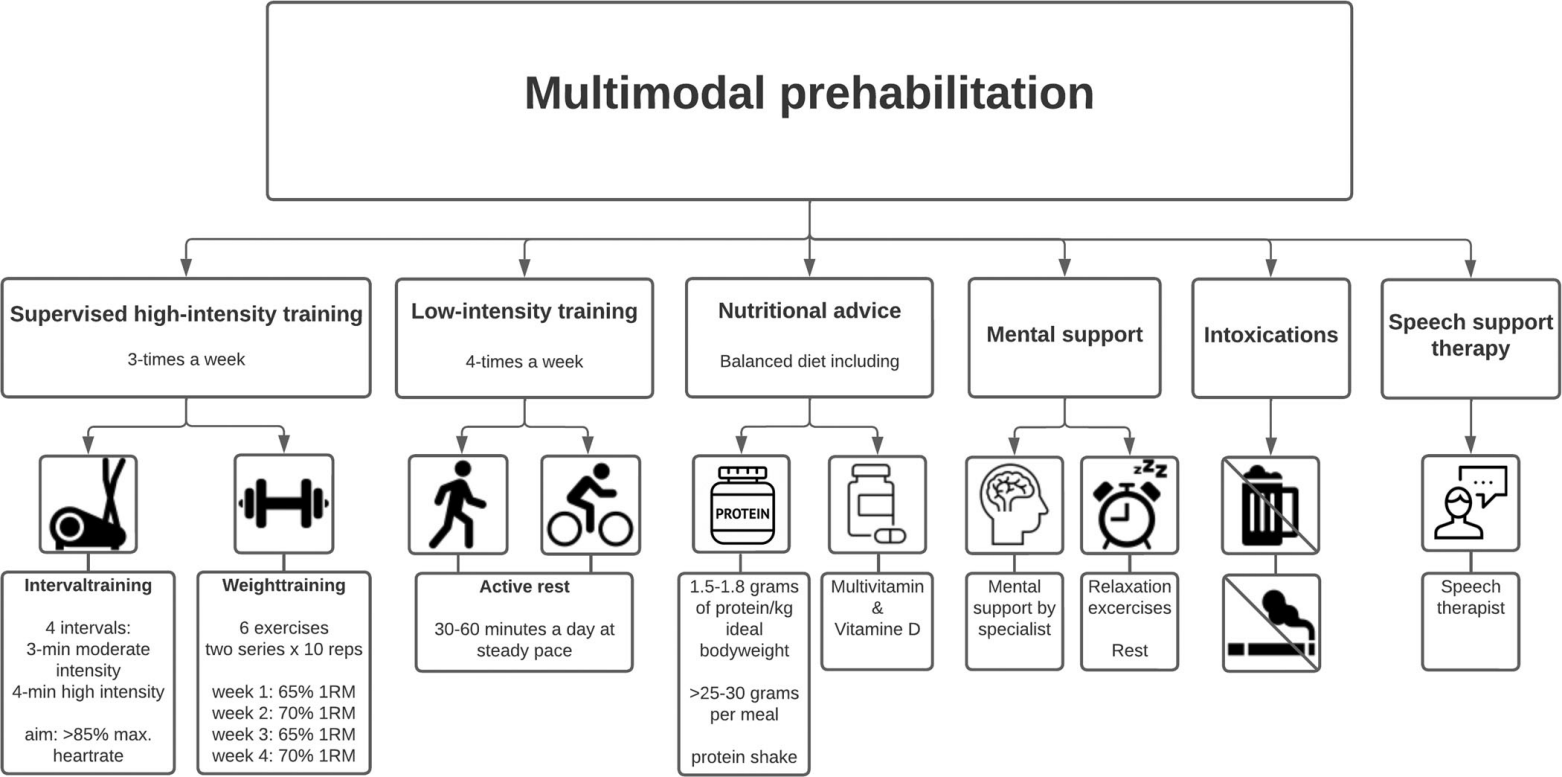
Multimodal prehabilitation.

Multimodal prehabilitation is composed of the following: supervised high‐intensity training (HIT), unsupervised low‐intensity training, a high‐protein diet, mental support and cessation of intoxications. Multimodal prehabilitation was conducted in line with our prior study and recommendations in other multimodal prehabilitation cancer studies [11, 27, 28]. To meet the specific needs of HNC surgery patients, specialised speech support therapy was added, as depicted in Figure 1.

At the oncology physiotherapist practice, supervised HIT was offered three times a week. Unsupervised low‐intensity training was done by the patient on the remaining days of the week. The supervised HIT consisted of interval and strength training. Based on functional capacity at baseline, the training programme was personalised accordingly. For more details, see Supporting Information A: Consensus on Exercise Reporting Template (CERT) [29].

A personalised high‐protein diet, containing at least 1.5–1.8 g protein per kg body weight a day and at least 25–30 g per meal, was made by a dietitian specialised in HNC patients. In case of a BMI less than 20 kg/m^2^ or more than 30 kg/m^2^, protein intake was corrected as recommended for cancer patients by the European Society for Clinical Nutrition and Metabolism [30]. Participants in the prehabilitation group were instructed to consume a whey protein shake (Nutri Whey Isolate, FrieslandCampina, Wageningen, the Netherlands) within 1 h after the supervised HIT and daily before bedtime. If achieving the daily protein intake through regular nutrition was not feasible, patients could incorporate an extra protein shake. Daily calorie intake was calculated with the World Health Organization (WHO) equation up to a BMI of 30 kg/m^2^ and with the Harris and Benedict equation in case of a BMI higher than 30 kg/m^2^ [31, 32, 33]. The outcomes of both calculations were increased by 30% to allow for physical exercise and HNC, as indicated by the national guideline [34]. Furthermore, each patient in the prehabilitation group was prescribed a multivitamin supplement of 50% of the recommended daily dose. Vitamin D was supplemented with 10 μg for participants younger than 70 years and 20 μg for participants aged 70 years or older [30, 34].

In case of mental distress, intensified guidance was provided by the case manager, a medical social worker or psychologist.

The guided smoking cessation programme SineFuma (Breda, the Netherlands) was offered to all active smoking participants. Patients were advised to cease alcohol consumption. Guidance by their own general practitioner took place if problematic alcohol use was present [35].

Speech support therapy consists of exercises to prevent swallowing and speech difficulties. Head and neck exercises are combined with swallowing exercises, which both are advised to execute at least three times daily [36].

### 2.7 Standard Care

All patients received standard perioperative care based on national guideline [37]. To date, no enhanced recovery after surgery (ERAS) protocol was implemented in our hospital.

### 2.8 Statistical Analysis

Data were analysed using SPSS Statistics, version 28 (IBM, Washington, DC). Normally distributed continuous variables were described in terms of means and standard deviations, and non‐normally distributed variables were described in terms of medians and interquartile ranges (IQRs). Comparisons were performed with a Student's *t*‐test, Mann–Whitney *U* test, Linear Mixed Models Analysis and Friedman Test as was deemed appropriate. Frequencies were expressed as percentages, and differences were analysed using the *χ* ^2^ test or Fisher's exact test where appropriate. A *p* value < 0.05 was considered significant.

## 3 Results

### 3.1 Patient Selection

50 patients were eligible for this study, of which 30 patients (60%) gave informed consent. The main reason for not participating was the travel distance to the associated specialised oncological physiotherapist in community practice.

### 3.2 Descriptive Data

Baseline characteristics, oncologic data and operative data are presented in Table 1. The mean age was 64.8 years, and most patients were classified as ASA 1 or 2 and could execute moderate to vigorous‐level activities. The most frequent surgical treatment was the commando procedure in 21 of 28 patients (75%).

**Table 1:** Baseline characteristics, oncologic data and operative data.

	Prehabilitation *n* = 28
Demographic data	
Age [years] ‡	64.8 (±15.9)
Sex ratio [F:M]	13:15
Body mass index [kg/m^2^] ‡	25.7 (±4.4)
ASA classification	
1–2	20 (71%)
3–4	8 (29%)
METs	
Light activity level (< 4)	4 (14%)
Moderate to vigorous level (> 4)	24 (86%)
Charlson comorbidity Index ‡	4.8 (±1.7)
History of radiotherapy	4 (14%)
Smoking	7 (25%)
Alcohol consumption	14 (50%)
Oncologic data	
Cancer type	
Oral and oropharyngeal cancer	26 (93%)
Salivary gland cancer	2 (7%)
Tumour size and extent	
T1	4 (14%)
T2	9 (32%)
T3	5 (18%)
T4a	10 (36%)
Lymph node involvement	
N0	18 (64%)
N1	3 (11%)
N2	5 (18%)
N3	2 (7%)
Type of surgery	
Commando procedure	21 (75%)
Reconstruction type	
Radialis	11 (39%)
Fibula	10 (36%)
Transoral resection with SNP	6 (21%)
Parotidectomy with neck dissection	1 (4%)

*Note:* Values are *n* (%), unless otherwise indicated; values are **‡** mean (SD); values are **†** median (IQR). Abbreviations: ASA, American Society of Anesthesiologists physical classification score; Commando procedure, combined mandibulectomy and neck dissection; METs, metabolic equivalent for task units; SNP, sentinel node procedure.

### 3.3 Primary Outcome: Feasibility

As stated in patient selection, the participation rate in this study was 60%, as 30 of 50 patients gave informed consent. Of those 30 patients, two patients were discontinuing prehabilitation based on medical reasons, leading to a dropout rate of 7%. The medical reason for one patient was admission to the hospital due to gastrointestinal disease, and the other patient reported feeling too weak to engage in prehabilitation.

The remaining 28 patients attended a mean of 8.6 (±2.2) supervised training sessions over a period of 23.5 (±5.7) days between the start of prehabilitation and surgery. One patient was unable to complete the required minimum of six sessions at the community physiotherapist practice due to a broken foot at the start of the supervised training. Thus, the adherence rate was 96%. No adverse events were reported during prehabilitation.

### 3.4 Secondary Outcomes: Functional Outcomes of Prehabilitation

At baseline, one patient had missing data concerning the 1RM chest press due to a low tolerance level. After completing the programme, 26 of 28 patients (93%) performed the functional tests 2–3 days preoperatively. Of the two patients missing preoperative testing, one was admitted early due to tumour‐related problems with nutrition and shortness of breath, and the other patient reported feeling unwell. The tests were repeated 6 weeks after surgery in 22 of 28 patients (79%). Reasons for not conducting the test included feeling too weak (*n* = 2), admission to a rehabilitation centre (*n* = 1), transportation issues (*n* = 2) and not being compliant (*n* = 1). Missing data was noted due to the inability to perform certain tests following a radialis reconstruction in 4 of the 22 patients (18%) for the 1RM lat pulldown, 3 of 22 patients (14%) for the 1RM bicep curl and 1RM chest press.

All functional tests showed improvements by prehabilitation, with the 6MWT being significant, all compared to baseline (*p* ≤ 0.05). Six weeks postoperatively, no statistically significant differences were observed in all tests compared to baseline. Despite this, all tests except for the steep ramp test showed increased outcomes. Notably, 12 of 22 patients (55%) showed improvement in the 6MWT, with 9 of them (32%) of more than 20 m 6 weeks postoperative compared to baseline. There was no statistically significant difference in attendance at supervised training between those who showed improvement (8.7 ± 2.2) and those who experienced a decrease (9.3 ± 2.6) in the 6MWT at 6 weeks postoperative compared to baseline. Results are presented in Table 2.

**Table 2:** Functional outcomes prehabilitation group.

	T0	T1 (2–3 days postop) compared to T0	*p*	T2 (6w postop) compared to T0	*p*
Sit‐to‐stand test [repetitions]	12.0	1.34	0.23	1.35	0.25
6MWT [m]	375.8	71.86	< 0.05*	13.99	0.71
Steep ramp test VO_2_ max [L/min]	1.3	0.07	0.41	−0.02	0.81
1RM tests					
Leg press [kg]	92.8	11.95	0.12	14.14	0.08
Latt pulldown [kg]	30.4	3.85	0.14	3.19	0.27
Chest press [kg]	34.4	4.37	0.26	2.08	0.62
Biceps curl [kg]	35.6	5.63	0.19	4.61	0.33

*Note:* Values T0 (baseline) are mean; values T1 (2–3 days preoperative) compared to T0 and values T2 (six weeks postoperative) compared to T0 are estimates.

Abbreviations: 6MWT, 6‐min walking test; 1RM, one repetition maximum.

### 3.5 Secondary Outcomes: Postoperative Outcomes

Overall, 30‐day postoperative complications occurred in 16 of 28 (57%) patients (see Table 3). Six major complications (Clavien–Dindo ≥ III) occurred in 6 of 28 patients (21%). After a commando procedure, 12 of 16 (75%) experienced a complication. When identifying patients at a higher risk for developing complications, a history of radiation therapy, diabetes mellitus and a commando procedure were identified. In 5 of 8 patients (63%) with a history of radiation therapy and in 4 of 5 diabetic patients (80%), a postoperative complication occurred.

**Table 3:** Postoperative complications and mortality.

	Prehabilitation *n* = 28
30‐day complications	16 (57%)
Clavien–Dindo Classification	
Grade I	6
Grade II	12
Grade IIIa^a^	3
Grade IIIb^a^	3
Grade IV^a^	0
Grade V^a^	0
Comprehensive complication index †	8.7 (±27.3)
Length of hospital stay [days] ‡	13.0 (±6.1)
30‐day readmissions	2 (7%)
30‐day mortality	0 (0%)

*Note:* Values are *n* (%), unless otherwise indicated; values are **‡** mean (SD); values are **†** median (IQR).

^a^ Clavien‐Dindo classification IIIa or higher was classified as a major complication

30‐day mortality was 0%.

Length of hospital stay was a mean of 13.0 days, and readmissions occurred in 2 patients (7%).

### 3.6 Nutritional Outcomes Prehabilitation

At baseline, the caloric intake of 27 of 28 patients (96%) was below the calculated needs. Similarly, protein intake was found to be insufficient in 25 of 28 patients (89%). During the telephone consultation 1 week after the start of the programme, no problems with adherence to the dietary plan were reported, except for two patients (7%) who forgot to take their protein shakes after training sessions.

The outcomes of all the nutritional tests after prehabilitation showed no statistically significant differences compared to baseline as presented in Table 4.

**Table 4:** Nutritional outcomes prehabilitation group.

	*N*	T0 = baseline	T1 = 2–3 days preoperative	*p* value
PG‐SGA †	28	2 ( ± 5)		
Weight [kg]	25	80.2 ( ± 17.4)	79.1 ( ± 17.4)	0.38
Body mass index [kg/m^2^]	25	25.6 ( ± 4.6)	25.8 ( ± 4.7)	0.10
Handgrip strength [kg]	24	32.1 ( ± 12.7)	33.2 ( ± 12.9)	0.17
Bioelectrical impedance analysis				
Lean body mass [kg]	21	53.0 ( ± 9.6)	52.7 ( ± 9.8)	0.33
Body fat [%]	21	33.2 ( ± 8.2)	33.8 ( ± 7.9)	0.19
Upper arm circumference [cm]	20	29.0 ( ± 3.5)	29.0 ( ± 3.0)	0.92

*Note:* Values are mean (SD), unless otherwise indicated; values are **†** median (IQR).

Abbreviation: PG‐SGA, Patient‐Generated Subjective Global Assessment.

### 3.7 Other Multimodal Prehabilitation Interventions

At baseline, 7 of 28 patients (25%) were current smokers, and 14 of 28 (50%) were currently drinking alcohol. After prehabilitation, 4 of 7 patients (57.1%) ceased smoking, and 9 of 14 (64.3%) stopped drinking alcohol. Regarding frailty, 4 of 12 patients (33.3%) of 70 years and older were screened by a geriatrician as frail and consequently, a comprehensive geriatric assessment was conducted. During the hospital stay, the geriatrician was co‐treating these patients. All patients received relaxing exercises from the physiotherapist, and all patients were guided by the case manager. In 4 of 28 patients (14.3%), a referral to a medical social worker or psychologist was made.

## 4 Conclusion

This feasibility study reports the first results to date of multimodal prehabilitation in HNC surgery. It shows a relatively low participation rate of 60%, but with a high adherence rate, low dropout and no adverse events. The study is mainly limited by the participation rate due to the travel time to the physiotherapists. All functional capacity tests improved by prehabilitation, with a significant increase in the 6MWT. Six weeks after surgery, all physical tests remained higher than baseline, except for the steep ramp test.

## 5 Discussion

Recent research on prehabilitation for HNC patients undergoing surgery predominantly focused on preoperative nutrition interventions [15]. Three studies reported positive effects of prehabilitation on the length of hospital stay, of which one demonstrated a significant reduction in postoperative complications [38, 39, 40]. The literature reveals significant variations in postoperative complication rates, and many studies in HNC surgery do not report the Comprehensive Complication Index. This study shows a relatively high percentage of postoperative complications compared to two other studies [39, 41]. This discrepancy can be potentially attributed to differences in the nature and extent of surgical procedures between the studies. Systematic reviews with meta‐analyses in other oncological disciplines, such as esophagogastric cancer [42] and lung [11] cancer, demonstrate that an improvement in functional outcomes has beneficial effects in reducing postoperative outcomes and length of hospital stay. The results of this study are promising with respect to the improvement in the physical condition of HNC patients before surgery, despite the restricted timeframe between diagnosis and surgery.

Several limitations must be kept in mind when assessing this study. The participation rate of 60%, mainly caused by travel distance/capacity to the training sessions, could potentially influence the outcomes of this study. Patients experienced a barrier to travelling for 30 min to the physiotherapist's practice three times a week. As future plans for further centralisation of oncological care in the Netherlands [43] and its impact on increased travel distance [44], this issue could even get worse. As in line with issues in adherence to treatment in HNC patients, participation in prehabilitation could be multifactorial influenced by the demanding treatment schedule and socio‐economic and psychological factors [45]. Although the participation rate is similar to that of other recent studies, it seems not to be caused by the attitude of patients towards prehabilitation, as they are willing to improve their health status before surgery [46, 47, 48, 49]. The high compliance and low dropout rate observed in our study are in line with these findings, underscoring the receptiveness of patients eventually participating in prehabilitation. Addressing improving participation rate, further expansion of a nationwide oncologic network of physiotherapists supporting prehabilitation could be of solution. Also, home‐based multimodal prehabilitation by an app could be a solution, as patients could participate from their own homes and no travelling needs to take place [50]. Lastly, in larger countries such as the United States, Canada, Germany and France, patients are more used to bridging larger distances to get care, so the issue could potentially be of less concern.

Another limitation is that the time to participate in prehabilitation is limited by the procession time, withstanding improving patient fitness. In the Netherlands, a required timeframe of 30 days from consultation to surgery is indicated, although no strong evidence is present indicating worse oncologic outcomes. A recent study debating this issue in colorectal cancer found no worse oncologic outcomes if a longer processing time is kept [51]. However, no data on HNC surgery is available regarding this issue. Despite these limitations, this study provides a basis for further research to tailor multimodal prehabilitation to the specific needs of HNC surgery patients.

### 5.1 Implications for Clinical Practice

Integrating prehabilitation into HNC surgery, particularly considering its significant morbidity rates, holds promise for mitigating the relatively high rates of complications and, consequently, the impactful influence on the quality of life. To enhance the programme's applicability and better facilitate multimodal prehabilitation in HNC surgery patients, a suggestion could be to use multiple locations for the supervised training sessions, possibly achieved by involving more affiliated specialised physiotherapists. Also, multimodal prehabilitation by an app could be of interest. Future research should be conducted to detect which form of multimodal prehabilitation is best feasible in HNC surgery patients, optimising participation rate and simultaneously making patients resilient to surgery. Especially in HNC surgery patients, as opposed to other cancer types, (supervised) exercises regarding mobility for head, neck and shoulders; swallowing; vocal function and maintaining speech function should be added. Tests should be added to monitor these parameters in these patients, next to strength and endurance tests, to assess the effectiveness of prehabilitation and its impact on recovery.

## Author Contributions

H.S., D.M. and E.B. did the conceptualisation, reviewing/editing and supervision. H.S. also did the funding acquisition. R.H. did reviewing/editing and supervision. F.D. did reviewing/editing. L.G. and C.V. did the data analysis with an epidemiologist, as well as the investigation and writing. L.G. also did the conceptualisation and methodology. All authors approved the final version of the manuscript.

## Ethics Statement

The ethical committee of the Academic Centre for Dentistry Amsterdam (ACTA) approved this study (2023‐52850).

## Conflicts of Interest

The authors declare no conflicts of interest.

### 1 Transparent Peer Review

The peer review history for this article is available at https://www.webofscience.com/api/gateway/wos/peer-review/10.1111/jhn.70047.

## Supporting information

Supporting information.

## Data Availability

The data that support the findings of this study are available on request from the corresponding author. The data are not publicly available due to privacy or ethical restrictions.
