# Supplementary material for: Multimodal Prehabilitation in Head and Neck Cancer Patients Undergoing Surgery: A Feasibility Study
Source: J Hum Nutr Diet. 2025 Mar 27;38(2):e70047. doi: 10.1111/jhn.70047 (PMC11950714; doi:10.1111/jhn.70047)
Supplement: Supplementary file 1 — Supporting information. [file JHN-38-0-s001.docx]

# Supplement A. CERT

| **Item category** | **Item no.** | **Abbreviated item description** |  |
| --- | --- | --- | --- |
| What: materials | 1 | Type of exercise equipment | Interval training; bicycle, treadmill, rowing machine, or other aerobic machines  Strength training; leg press, chest press, biceps curl, lat pull down, low row and the back trainer |
| Who:  provider | 2 | Qualifications, teaching/supervising expertise, and/or training of the exercise instructor | Physiotherapist specialized in oncological patients |
| How:  delivery | 3 | Whether exercises are performed individually or in a group | Exercises are performed individually due to the fact that patients all have their own prehabilitation time schedule |
|  | 4 | Whether exercises are supervised or unsupervised | The interval and strength training is supervised. The low intensity training is at home unsupervised |
|  | 5 | Measurement and reporting of adherence to exercise | The 1RM was taken from four strength exercises (leg press, lat pulldown, biceps curl, chest press) were done at two seconds of concentric force and two seconds of eccentric force, after which the 1RM was calculated with the Brzycki formula: 1RM= W*36/(37 –r) (W = weight in kg, r = repetitions) [52].  The STS was performed as the maximum number of repetitions within a period of 30 seconds.  The 6MWT is defined as the maximum walking distance (in meter) in six minutes [53].  The maximum rate of oxygen consumption (VO_2_max) was calculated using the SRT (VO_2_-max in (L/min) = 0,0067 * WmaxSteepRamp + 0,3583) [54,55].  Patients were in charge of their own adherence reporting using a daily logbook |
|  | 6 | Details of motivation strategies | Because of the supervised element three times a week patients got external motivated by the physiotherapists. By giving them a big part in their recovery, we aimed to establish some intrinsic motivation. |
|  | 7 | Decision rules for progressing the exercise program | The supervised HIT consisted of interval and strength training. Based on functional capacity at baseline the training program was accordingly personalized. During the period of supervised HIT, an aim increase of 10% in VO2max and 1RM was pursued.  The interval training aims to reach Borg levels 15-17 and >85% of the maximum heart rate (measured during the SRT).  The weight and/or resistance used in the program was individualized according to the calculated 1RM results at baseline. In week 1 the training was started at 65% of the 1RM, which was weekly increased to 70% at week 2 and 75% at week 3). If the last 10 reps were not feasible the weight or resistance was adjusted by 5-10% less in the subsequent workout if it was considered too easy the resistance or weight was increased by 5-10% in the subsequent training |
|  | 8 | Each exercise is described so that it can be replicated (eg illustrations, photographs) | leg press; To start, sit with your back against a padded backrest and your feet on two large footrests. Your knees are bent to start the exercise. To move the weight, you must straighten your legs and then return them to the bent position  chest press; sit down in the seat and place the back of your arms on the pad in front of you. Grab the handles with you palms facing upwards. Now curl the handles up to your shoulders and then lower them back down. This completes one repetition  lat pull down; Adjust the pad so it sits snugly on your thighs to minimise movement. Grasp the bar with a wide grip, looking forward with your torso upright. Retract your shoulder blades and pull the bar down in front of you to your upper chest. Squeeze your lats at the bottom of the move. Resist the temptation to lean back to aid the movement.  Low row; adjust the seat height, sit down and grab the handles. Start with your arms extended, pull straight back by squeezing your lats. Control the weight back to the starting position. Repeat the desired number of repetitions.  Low-intensity training was a 30-60min exercise like walking or cycling. |
|  | 9 | Content of any home program component | The at home component consists of low-intensity training (30-60min exercise like walking/cycling) 4-times a week continues 3 days prior to surgery. |
|  | 10 | Nonexercise components | - |
|  | 11 | How adverse events that occur during exercise are documented and managed | The physiotherapists documented adverse events during exercise and reported it back to the research team. If medical referral or adaptation in the training schedule was necessary, this would be provided. |
| Where; location | 12 | Setting in which exercises are performed | Supervised training at a physiotherapist practice |
| When, How much; dosage | 13 | Detailed description of the exercises (eg sets, repetitions, duration, intensity) | Interval training; has a duration of 28 minutes (min) (4 intervals of 3-min moderate intensity, 4-min high intensity.  Strength training consisted of two series including 6 exercises of 10 repetitions (reps) each (leg press, chest press, biceps curl, latt pull down, low row and the back trainer). |
| Tailoring; what, how | 14 | Whether exercises are generic (“one size fits all”) or tailored to the individual | All exercises are generic but will be adjusted in intensity for the individual. |
|  | 15 | Decision rule that determines the starting level of exercise | All patients will do the functional baseline tests (VO2max, sit to stand, 6 minute walking test) and will be adjusted according to item no 7 |
| How well; planned, actual | 16 | Whether the exercise intervention is delivered and performed as planned | Unfortunately, there was missing data for six patients at baseline due to a low tolerance level. The adherence level was lower as we expected with only 60% recorded their daily activity in their logbook. |

# Supplement B. Multimodal prehabilitation measurement points scheme

| Prehabilitation scheme | | | | | | |
| --- | --- | --- | --- | --- | --- | --- |
|  | | Preoperative | | | Surgery | Postoperative |
| Week | -4 | | -4/-3 | -1 | 0 | 6 |
|  | Inclusion | | Baseline (T0) | Preoperative (T1) |  | 6 weeks follow-up (T2) |
| Surgeon  / Case manager | Informed consent | | Distress thermometer | Distress thermometer | - | Distress thermometer |
| Physiotherapist | - | | SRT  1RM  STS  6MWT | SRT  1RM  STS  6MWT | - | SRT  1RM  STS  6MWT |
| Dietician | - | | BMI  BIA  Hand grip strength  Upper arm circ.  PG-SGA | BMI  BIA  Hand grip strength  Upper arm circ. | - | - |
| Researcher | - | |  | Compliance | - | Complications  Mortality  LoS  Readmission |

SRT steep ramp test; 1 RM one repetition maximum; STS sit-to-stand test; 6MWT six-minute walking test; BMI body mass index; PG-SGA Patient-Generated Subjective

**Reference**

52. M. Brzycki, “Strength Testing—Predicting a One‐Rep Max From Reps‐to‐Fatigue,” Journal of Physical Education, Recreation & Dance 64 (1993): 88–90, https://doi.org/10.1080/07303084.1993.10606684.

53. ATS Committee on Proficiency Standards for Clinical Pulmonary Function Laboratories, “ATS Statement: Guidelines for the Six‐Minute Walk Test,” American Journal of Respiratory and Critical Care Medicine 166 (2002): 111–117, https://doi.org/10.1164/ajrccm.166.1.at1102.

54. K. Meyer, L. Samek, M. Schwaibold, et al., “Physical Responses to Different Modes of Interval Exercise in Patients With Chronic Heart Failure—Application to Exercise Training,” European Heart Journal 17 (1996): 1040–1047, https://doi.org/10.1093/oxfordjournals.eurheartj.a015000.

55. A. T. R. Weemaes, M. Beelen, B. C. Bongers, M. P. Weijenberg, and A. F. Lenssen, “Criterion Validity and Responsiveness of the Steep Ramp Test to Evaluate Aerobic Capacity in Survivors of Cancer Participating in a Supervised Exercise Rehabilitation Program,” Archives of Physical Medicine and Rehabilitation 102 (2021): 2150–2156, https://doi.org/10.1016/j.apmr.2021.04.016.
